# An Introduction to Costing and the Types of Costs Used within Health Economic Studies

**DOI:** 10.1007/s41669-025-00602-1

**Published:** 2025-10-20

**Authors:** Hugo C. Turner, Juan Carlos Rivillas-Garcia, Shankar Prinja, Trinh Manh Hung, Saudamini Vishwanath Dabak, Brian A. Asare, Mark Jit, Yot Teerawattananon

**Affiliations:** 1 MRC Centre for Global Infectious Disease Analysis, School of Public Health, Imperial College London, London, UK; 2 School of Public Health, Department of Epidemiology and Biostatistics, MRC Centre Environment and Health, Imperial College London, London, UK; 3 Department of Community Medicine and School of Public Health, Postgraduate Institute of Medical Education and Research, Chandigarh, India; 4 School of Public Health, Faculty of Health, Medicine and Behavioural Science, The University of Queensland, Brisbane, Australia; 5 Health Intervention and Technology Assessment Program Foundation (HITAP), Nonthaburi, Thailand; 6 Health Technology Assessment Unit, Ministry of Health, Accra, Ghana; 7 Department of Infectious Disease Epidemiology, Faculty of Epidemiology and Population Health, London School of Hygiene and Tropical Medicine, London, UK; 8 Department of Global and Environmental Health, School of Global Public Health, New York University, New York, NY USA; 9 Saw Swee Hock School of Public Health, National University of Singapore (NUS), Singapore, Singapore

## Abstract

The number of published health economic analyses, especially economic evaluations, has rapidly expanded globally since the 1990s, and costs are an essential component of such studies. Cost is a general term that refers to the value of the resources/inputs used to produce a good or service. However, within health economics, there are several different types of costs (such as financial, economic, unit, average, etc.). The terminology and application of these cost types often differ, leading to inconsistencies in the health economics literature. These inconsistencies create challenges in comparing studies and hinder the use of health economic analyses to effectively inform policy decisions. This paper aims to provide an up-to-date overview of the cost types, key cost terms, and definitions of different cost measures used within health economics, while highlighting key inconsistencies in the literature. We also discuss common adjustments made to cost data, such as accounting for inflation, discounting, and currency conversions, as well as the influence of economies of scale and scope on cost estimates. We highlight that the different definitions/categories for the different types of costs are not mutually exclusive and that the type of cost that should be used will depend on the purpose of the study, highlighting recommendations of what to do in practice where relevant. The content was tailored to be relevant across both high-income and LMIC contexts.

## Key Points for Decision Makers

**Table:** 

Health economic evaluations have grown significantly since the 1990s, but inconsistencies in cost terminology and application can at times hinder comparability and policy relevance.
This paper reviews key cost types and definitions in health economics, discusses common cost adjustments, and offers practical recommendations to improve clarity and consistency in economic analyses.
We emphasize that the various definitions and categories of cost types often overlap, and selecting the appropriate cost type should be guided by the specific objectives of the study.

## Background

The number of health economic analyses published, especially economic evaluations, has rapidly expanded globally since the 1990s [1, 2]. Cost analyses are an essential component of most of these studies. For example, they are used to investigate the cost of health interventions as well as the broader economic burden that a specific disease places on society (cost-of-illness analyses [3]). Cost data are a vital component of full economic evaluations [4] as well as other types of economic analysis, such as budget impact analyses [5]. Increasingly, cost data are used to inform copayment rates under government-funded health insurance schemes [6, 7]. Additionally, intervention cost data play a crucial role in informing price negotiations between payers and manufacturers [8].

“Cost” is a general term that refers to the value of resources/inputs used to produce a good or service (usually expressed in monetary terms). Within health economics, a variety of (nonmutually exclusive) terms are used to describe costs (such as financial, economic, unit, and average, etc.) depending on the context of the study (Box 1). However, inconsistencies in terminology and usage across the field have led to discrepancies in the health economic literature, making it challenging to standardize definitions and compare findings [9, 10].

The inconsistency and overlapping boundaries between terms and their uses create a barrier to comparing results across studies and using health economic studies to inform policy decisions. For example, it has been reported that the lack of standardization in terminology, implementation, and principles for vaccine delivery costing reduced the potential for using economic evidence across studies for informing immunization program policy [21]. Concerningly, a misunderstanding of costing terminology and approaches can lead to inefficient resource allocation decisions [23].

Previously, we wrote about the concepts surrounding economic costs [14], as well as providing an overview of the perspectives used in health economic analysis (which influences what costs will be included) [24]. This paper complements these by providing a more general introductory overview of the main types of costs used within health economics targeted to a broad audience. This is relevant as, although costs are a fundamental concept within health economic analysis, they are currently inconsistently defined within the literature [9], and there is notable variation in the methods and reporting of costs for global health interventions [11]. Furthermore, capacity in health economics has been identified as an ongoing issue, particularly for many low- and middle-income country (LMIC) settings [25–27]. There is therefore a need for more open access introductory texts covering key concepts relating to health economic studies—particularly within a global health context also considering LMICs and not only focusing on high-income settings.

This paper builds on and complements previous texts in this area (such as refs. [9–12, 19, 21, 22, 28, 29]. We provide a broader, more introductory overview of cost types, key terminology, and common adjustments, highlighting key inconsistencies in the literature. This is intended for a wider audience, including practitioners and policymakers with limited health economics experience. It is hoped that providing this overview guidance on the types of costs will increase the consistency of future health economic studies and act as a precursor for those with limited health economic experience to the more technical guidance already available [30].

To inform the writing of this paper, we conducted a targeted narrative review. Core terminology was drawn primarily from the Global Health Cost Consortium Reference Case [11], which harmonized costing definitions through an extensive review of existing guidelines and a global expert consensus process. When needed, additional terms were sourced from alternative references, identified through the repository of supplemental sources on the Global Health Cost Consortium website [30] and other relevant guidelines/sources known to the coauthors (Boxes 1 and 2). All definitions were further refined through coauthor discussions to ensure that they were clear, consistent, and applicable across both high-income and LMIC contexts.

**Box 1:** Glossary summarizing key types of costs

Capital costs: these are one-time costs for items that have a useful life of more than 1 year (such as buildings, vehicles, or medical equipment) [11]
Charge: represents the amount a service provider “bills” for services (such as what is on the patient’s bill from a hospital) [12]
Direct costs: represent costs directly associated with the resources used for the treatment/management of a disease or health condition. These can be stratified into direct medical costs and direct nonmedical costs [13]
Direct medical costs**:** represent the costs related to the use of medical resources/goods/services (such as the costs associated with healthcare services, diagnostic tests, drugs, etc.)
Direct nonmedical costs**:** represent the costs related to the use of nonmedical resources or costs associated with obtaining healthcare services (such as costs related to the patient’s/caregiver’s travel, food, etc.)
Economic cost: represents the full value of the resources utilized in providing an intervention. Crucially, they are intended to capture the resources’ opportunity cost, and they are based on the value of the resources’ next-best alternative use that has been forgone owing to them being utilized and not simply the monetary amount paid for them [14]
Financial cost: represents the actual financial outlays for the goods, resources, and services that are purchased [14]
Fixed costs: costs that do not vary with scale (i.e., they do not change with the level of output) [11]
Future related medical costs: medical costs occurring in the future that are directly related to the disease under investigation. For example, an intervention to treat heart disease will extend a patient’s life, but there will be future costs associated with routine visits to a cardiologist [15]
Future unrelated medical costs: medical costs that are unrelated to the disease being investigated. For example, an intervention may extend a patient’s life, but they may then subsequently incur other medical costs associated with unrelated conditions [15]
Future unrelated nonmedical costs: future costs that are not related to medical resources. This can include the monetary value of the productivity gains associated with an intervention [15]
Incremental costs: the difference in costs between two competing interventions or strategies [11]
Indirect cost: represents costs not directly associated with the provision of healthcare services but incurred as a consequence of an illness or intervention [13]
Intangible costs: describe costs that cannot be directly quantified in monetary terms (such as costs related to pain or depression as a result of illness or treatment) [16, 17]
Marginal costs: the additional cost incurred by producing one extra unit of output or delivering one more service [11]
Opportunity costs: the opportunity cost of making a particular choice is the value of the next-best alternative that is forgone [14]
Out-of-pocket payments: spending directly incurred by an individual/household for health services that are not reimbursed by any third party [18]
Productivity costs (a subtype of indirect costs): incurred when the productivity of individuals (patients and caregivers) is affected by the illness/disability and/or the intervention in question. They represent the monetized value of such productivity losses and can include both paid and unpaid work [19]
Recurrent costs: costs for the resources/inputs with useful lives of less than 1 year. This includes supplies and personnel [11]
Reimbursement rate (tariffs): the set monetary amount healthcare systems pay a health service provider for particular categories of activity/tasks (such as the amount of money a hospital would receive from a governmental social health insurance program for performing a diagnostic test). In contrast, this term can also be used to describe the amount an insured person is reimbursed by their insurance company after having incurred a healthcare-related expense
Shared (joint) costs: relate to resources that are jointly used between different interventions [20]
Start-up costs: relate to initial one-time programmatic activities, such as initial training activities, and initial sensitization/social mobilization [21]
Sunk cost: costs that have already been incurred and that cannot be retrieved [11]
Transfer costs: transfer costs or payments are financial flows from one part of society to another, that do not consume resources but simply transfer the power to use resources from one person or sector to another [22]
Variable costs: costs that vary with scale (i.e., they change with the level of output of an intervention) [11]

**Box 2:** Relevant guidelines, papers and reference cases related to costing

Note that these were identified on the basis of what the coauthors commonly utilize in their research, and it is not intended to be an all-exhaustive list. A more comprehensive list of key sources for guidance on costing methods can be found on the Global Health Cost Consortium website [30]
1. Global Health Cost Consortium: Reference case for estimating the costs of global health services and interventions [11]
2. UNAIDS: Costing guidelines for HIV prevention strategies [31]
3. Hutton G, Baltussen R: Cost valuation in resource-poor settings [22]
4. WHO-CHOICE: WHO’s guide to cost-effectiveness analysis [20] 5. Levin A, Boonstoppel L, Brenzel L, Griffiths U, Hutubessy R, Jit M, Mogasale V, Pallas S, Resch
6. S, Suharlim C, Yeung KHT: WHO-led consensus statement on vaccine delivery costing: process, methods, and findings [21]
7. World Health Organization: Costs, standard terminology and principles for vaccine delivery [32]
8. Brenzel L: Common approach for the costing and financing of routine immunization and new vaccines (EPIC) [28]
9. Janowitz B, Bratt JH: Methods for costing family planning services [33]
10. Culyer AJ: Cost, context, and decisions in health economics and health technology assessment [34]
11. Jeet G, Masaki E, Vassall A, Prinja S: Costing of essential health service packages: a systematic review of methods from developing economies [13]
12. ISPOR Drug Cost Task Force: Good research practices for measuring drug costs in cost effectiveness analyses—Parts I–VI [35–40]

## Methods/Approaches of Costing

**Fig. 1:**
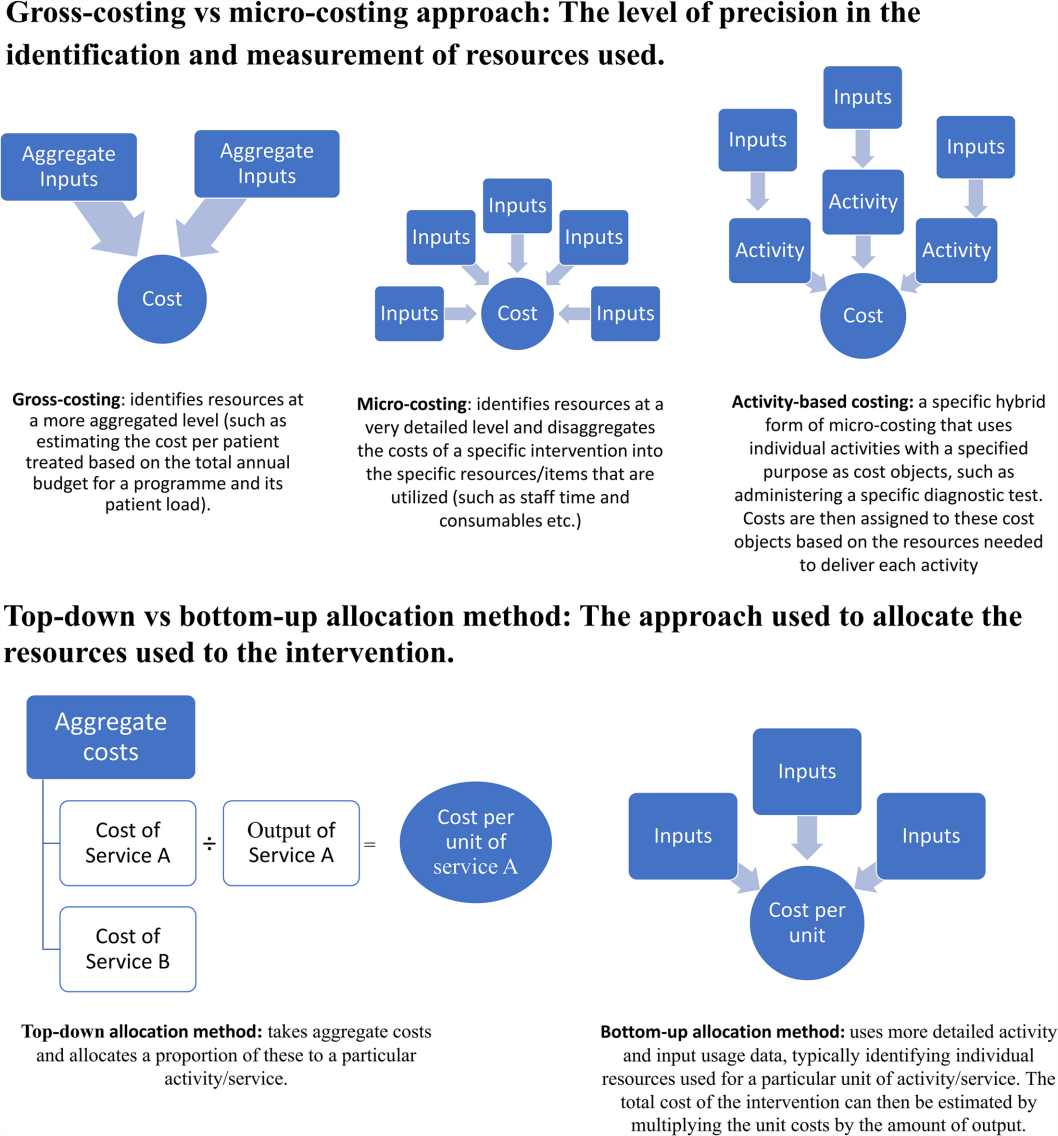
Types of costing terminology

There are different types of costing methods and they are typically classified by two key sets of classifications: bottom-up versus top-down and microcosting versus gross-costing (Fig. 1) [29]. Terminology to describe costing methods is currently used inconsistently in the literature, and some of these terms are sometimes used interchangeably [11]—however, there can be a formal difference between these groups of terms [9, 10, 29].

The terms gross versus microcosting refers to the level of precision/disaggregation in the identification and measurement of resource and cost components [9] (Fig. 1). A gross-costing approach is a method of identifying the cost of the resources utilized at a more aggregated level (such as estimating the cost per patient treated on the basis of the total annual budget for a treatment program and its patient load). This approach can be useful for initial cost assessment or when detailed data are not available. In contrast, the microcosting approach involves identifying resources at a very detailed level and disaggregates the costs of a specific intervention into the specific individual resources/items that are utilized (i.e., the analyst aims to estimate the usage of each input separately, such as staff time, drugs, consumables, etc.) [9, 11]—this is also referred to as an “ingredient-based” approach*.* This will normally be undertaken at the service level. Microcostings are generally more comprehensive and capture more input usage, while gross-costings tend to underestimate costs but may be more feasible when access to data or time for conducting a study is limited [11, 41].

An additional approach is activity-based costing (as well as its simplified form time-driven activity-based costing), a specific hybrid form of microcosting [9, 11]. It is based on the concept that activities consume resources and that the outputs of health interventions are the result of such activities*.* Therefore, if the resource consumption of activities can be measured more accurately, a more accurate cost estimate can be calculated [12]. An activity-based costing uses individual activities with a specified purpose as cost objects, such as administering a specific diagnostic test. Costs are then assigned to these cost objects on the basis of the resources required to deliver each activity [12]. This approach was developed to improve the accuracy of unit cost estimates, particularly related to how overhead costs are assigned [9].

In contrast, the terms, “bottom-up” and “top-down” refer to the way in which a resource is allocated to the cost of the intervention being estimated [11]. A top-down costing approach takes aggregate costs and allocates a proportion of these to a particular activity/service [9, 42]. In contrast, the bottom-up costing approach uses more detailed activity and input usage data, typically identifying individual resources used for a particular unit of activity/service [9, 11, 42]. The total cost of the intervention can then be estimated by multiplying the unit costs by the amount of output (e.g., the unit cost of vaccination multiplied by the number of people vaccinated). Using the top-down approach may capture inputs for which the usage cannot be observed or that are affected by seasonal factors [11]. It may also better capture inefficiencies and wastage [11]. In comparison, bottom-up approaches may allow an improved understanding of service provision and may also be better at characterizing variation in practice [43].

In practice, a gross-costing typically uses the top-down application approach whereas microcosting typically uses the bottom-up approach [11]. However, this is not universal, and the distinction between these terms can be a question of degree. For example, within a microcosting study, even if the bottom-up approach is predominantly being used at the service level, the top-down approach may also be used to allocate certain inputs from higher levels (such as administrative overhead or programmatic costs incurred at the national level) [11].

A further distinction to the type of costing is whether the study is aiming to reflect the cost of “normative best practice” (i.e., what is in treatment guidelines) or the costs of implementing an intervention in the “real world” (which may include inefficiencies or exclude certain cost components) [11]. It is important to note that the difference between these (as well as which is higher or lower) will depend on the context of the setting. In practice, this will often not be a precise division, and studies may have elements of both [11].

Finally, the costing may involve retrospective or prospective data collection [21]. Retrospective data collection involves data collection after resource use has been completed. In contrast, prospective data collection involves the direct observation of resource use during intervention implementation [21]. There are also projected costs that are estimated before an intervention is introduced.

These different approaches will likely yield different cost estimates, and their use will depend on factors such as resource availability, the timeframe, and the fit for purpose/research question.

## Key Types of Costs

There are a variety of different ways to classify costs within health economics. It is important to consider two key points: (1) the different definitions/categories for the different types of costs are not mutually exclusive or necessarily additive and (2) there can be variation and inconsistencies in how the definitions are used in practice.

### Direct Costs

Direct costs represent costs directly associated with the resources used for the treatment/management of a disease or health condition. Direct costs are further split into direct medical and direct nonmedical costs (Table 1). Direct medical costs refer to the costs related to the use of medical resources/goods/services (such as diagnostic tests, drugs, etc.). Direct nonmedical costs represent the costs related to the use of nonmedical resources (such as costs related to the patient’s/caregiver’s travel and food).

**Table 1:** Classification of direct medical, direct nonmedical, and productivity costs/indirect costs

Cost type	Examples
**Direct costs: represent costs directly associated with the resources used for the treatment/management of a disease or health condition**	
Direct medical costs: include the value of goods and resources directly related to the provision of medical services	Physician services, diagnostic tests, drugs, surgical procedures, etc.
Direct nonmedical costs: include the value of resources related to the consumption of nonmedical resources but not directly related to the provision of medical service	Transportation costs to healthcare facilities, food expenses, accommodation, childcare, paid caregivers, etc.
**Indirect costs: represent costs not directly associated with the provision of healthcare services but incurred as a consequence of illness or intervention**	
Productivity costs: represent the value of the productivity losses that result from the direct impact of illness, treatment, or premature death and/or caregiving for the patient.	Missed paid and unpaid work by the patient and their unpaid informal caregivers^1^
Broader costs: represent the broader types of indirect costs beyond the productivity costs defined above	The longer-term broader productivity losses that result from the reduced economic value of a worker’s experience/skills (such as from the lasting effects of disrupted schooling or impaired cognitive development), the loss of productive assets as a result of financing catastrophic medical expenditure, broader macroeconomic losses as a result of disruptions to labor or demand, and impacts on other sectors (such as reduced tourism due to the risk of infectious disease transmission)

^1^There is variation in the literature regarding whether the valuation of informal caregivers’ time is included as a productivity cost or a direct nonmedical cost

### Indirect Costs and Productivity Costs

Indirect costs represent costs that are not directly related to the provision of healthcare services. One of the most important subtypes of indirect costs is productivity costs (Table 1). These occur when the productivity of individuals is affected by the illness/disability and/or the intervention in question [19] and represent the monetized value of such productivity losses. It is important to note that there is variation in how the indirect-cost term is applied, and in accounting/business disciplines, “indirect” costs relate to the supporting/overhead activities [9]. Even within the health economic literature, there is variation in the use of terminology in this area. Consequently, within the health economics field, there is a move toward the use of the term productivity costs instead of indirect costs—to avoid confusion [44, 45]. These typically represent the value of the productivity losses that result the direct impact of illness, treatment, or premature death, i.e., number of lost workdays due to illness. That said, the term indirect costs could also include broader types of relevant indirect costs such as those relating to other sectors, longer-term broader productivity losses that result from the reduced economic value of a worker’s experience/skills, and macroeconomic losses (Table 1).

Productivity costs can be associated with an individual accessing an intervention—such as valuing the time an individual spends going to a health clinic to get a vaccine. They can also relate to the productivity losses associated with a disease more broadly (the number of days they cannot perform paid/unpaid work owing to illness or premature death). There is variation regarding whether the valuation of informal caregivers’ time is included as a productivity cost or a direct nonmedical cost [46]. The key principles related to productivity costs are outlined in the following texts [4, 19, 44, 47, 48].

**Table 2:** Description of key terms related to productivity cost calculations (partly adapted from ref. [19])

**Terms related to the measurement of productivity losses**	
Absenteeism	Productivity losses related to not attending work (i.e., missed days of work)
Presenteeism	Productivity losses related to diminished functioning in terms of quantity and/or quality while attending work
Unpaid work	Productive activities outside of the official labor market (such as time spent on housework, taking care of children, voluntary work, and unpaid caregiving)
Multiplier effects	The effects on overall or “team output” due to absenteeism or presenteeism of an ill worker—i.e., when the reduced productivity of an individual negatively affects the productivity of others
Compensation mechanisms	The ways in which lost productivity is compensated for
Lost workdays	The number of days an individual is unable to work owing to illness or disability
**Theoretical underpinning**	
Capturing lost compensated labor	Productivity costs are only intended to capture working time lost from the labor system. These only quantify the actual productivity losses from the lost work from those of working age. Under this underpinning, the productivity losses are often valued on the basis of the actual income of the sample or by using age or sex-specific wage rates
Capturing lost time	Productivity costs (also referred to as time costs) are intended to capture the opportunity cost of lost productivity more broadly by considering any time lost and not just actual lost working time (also potentially including lost leisure time). With this concept, the calculations may value productivity losses the same way for the whole sample using an average value (such as using the average income or a daily per capita gross domestic product)
**Valuation of productivity losses**	
Human-capital approach	Under this approach, all potential production not performed by a person because of morbidity or early mortality is counted as a production loss
Friction-cost approach	Under this approach, the calculated production losses are limited to the time needed to replace an ill employee and train a new employee (friction period [49]) i.e., only the productivity lost by an ill employee before they are replaced
**Valuation of unpaid work**	
Opportunity-cost approach	Measures the value of time spent on unpaid work in terms of the value of the next best alternative activity the individual has forgone to perform it—such as valuing the unpaid work performed by an informal caregiver on the basis of what they could have earned in terms of paid employment
Replacement-cost/proxy cost approach	Measures the value of time spent on unpaid work on the basis of what it would cost to hire a paid worker to perform the same tasks—such as valuing the unpaid work performed by informal caregivers on the basis of what it would cost to hire someone to perform those tasks
Willingness-to-pay approaches	The unpaid work performed by informal caregivers can be valued using contingent valuation approaches. This derives a monetary value by asking respondents what their minimum compensation would be to provide an extra hour of informal care (willingness to accept) or the maximum amount they would be willing to pay to perform 1 hour less of informal care (willingness to pay) [48]. Other willingness-to-pay approaches can also be used (such as conjoint analysis) [48]

There is significant variation in how productivity costs are quantified, and there are several ongoing areas of debate [4, 50, 51]. This is owing to variations in both how productivity losses are qualified and how they are subsequently monetized. This can lead to inconsistencies between studies [4, 52].

Quantifying productivity losses accurately is challenging, and the different approaches used can give markedly different outcomes [4]. For instance, the human-capital approach can give notable different (and usually much larger) results to the friction-cost approach (Table 2) [44, 47, 53]. Furthermore, the majority of studies focus on quantifying losses associated with absenteeism (i.e., missed days of work) whereas presenteeism (diminished functioning in terms of quantity and/or quality of while attending work) is often not accounted for [54]. The quantification of lost unpaid work is a neglected area [51, 55, 56]. What constitutes unpaid work and distinguishing it from leisure time remains a challenging area [19]. Reid’s “third person criterion” [57] proposes that only activities replaceable by a third person should be considered unpaid work, while other activities should be considered leisure. However, even when applying this, the distinction between leisure and unpaid work remains difficult in practice [55]. It should be noted that some argue that lost leisure should be included within productivity losses [58, 59].

It is important to consider that productivity losses can be influenced by compensation mechanisms (ways in which lost productivity is compensated for) as well as multiplier effects (where losses related to the effects of an ill employee’s diminished productivity negatively impact coworkers’ productivity) [19]. Brouwer et al. [60] highlighted that compensation mechanisms and multiplier effects are common and may substantially affect production losses. That said, they are often not accounted for.

In addition to how the productivity losses are quantified, the methodology of how they are valued/monetized is also variable [4, 19]. For example, there is variability in the wage source used to monetize productivity losses and how it is adjusted [4, 61].

This variation in the quantification of productivity losses and their valuation is, in part, linked to two contrasting theoretical underpinnings of productivity cost calculations (Table 2). One approach considers only the working time lost from the labor system. Within this, only actual productivity losses from the work of the working age group are considered and often valued on the basis of the sample’s actual income. Such calculations are more likely to make adjustments for labor force participation and/or use age or sex-specific wage data. In contrast, another approach captures the opportunity cost of lost productivity more broadly by considering any time lost (which can also include nonwork time). Similar to the concept of economic cost (described in a subsequent section [14]), such calculations aim to reflect the full value of the lost time to society—regardless of what productivity/wages are actually lost. These calculations sometimes value productivity/time losses the same way for the whole sample using a macroeconomic indicator (such as using the daily per capita gross domestic product [GDP] or gross national income [GNI] or national average wage value [44]). Although capturing lost time results in the productivity costs being more hypothetical (and less fiscal), there is arguably an advantage from an equity standpoint, as they avoid valuing productivity losses from groups of the population lower than others (e.g., the young, the old, and women). This variation in approaches needs to be considered when comparing studies and it is important to note that studies may not fit within this dichotomy, using elements of both approaches. In addition, further guidance is needed regarding the valuation of unpaid work, informal care, and leisure time [55, 62–64].

In addition to the methods being variable, the assumptions surrounding productivity cost calculations are often poorly reported. In Table 3, we present recommendations regarding this topic.

**Table 3:** Recommendations for reporting productivity cost calculations

**Quantification of productivity losses**
• Clearly report the approach to the productivity cost calculations (valuing lost labor versus lost time)
• Clearly describe and justify the method used to quantify productivity losses (e.g., absenteeism, presenteeism, production loss, etc.)
• Where possible, stratify results to capture the different types of productivity losses
• State if the human-capital approach or friction-cost approach is being used
• State if any ages are being excluded from the calculations—such as the productivity losses are only considered up to the retirement age (versus the average life expectancy)
• Acknowledge any limitations of the chosen method, and discuss potential biases
**Valuation of productivity losses**
• Clearly report and justify the wage source being used—as well as the unit value
• Outline any adjustments (such as the number of workdays assumed to convert a monthly or yearly income value to the corresponding daily value)
• Outline the valuation of unpaid work—justify the approach used
• Where possible, stratify results by the productivity costs related to patients and caregivers
• If possible, perform a sensitivity analysis

It should be noted that there is variation regarding the types of productivity costs included within economic evaluations, even when a societal perspective is used, and studies may show their results both with and without productivity costs or only include certain types of productivity costs (such as excluding those related to excess mortality) [24].

While the traditional classification of costs into direct and indirect categories remains widely used, there are other classification frameworks. For example, a sector-based approach would categorize costs according to the sector in which they fall (e.g., healthcare, household, and other societal sectors) [65]. This approach can improve clarity by explicitly linking costs to their origin, thereby avoiding some of the ambiguity surrounding direct/indirect cost classifications (Table 1).

### Financial versus Economic Costs

**Fig. 2:**
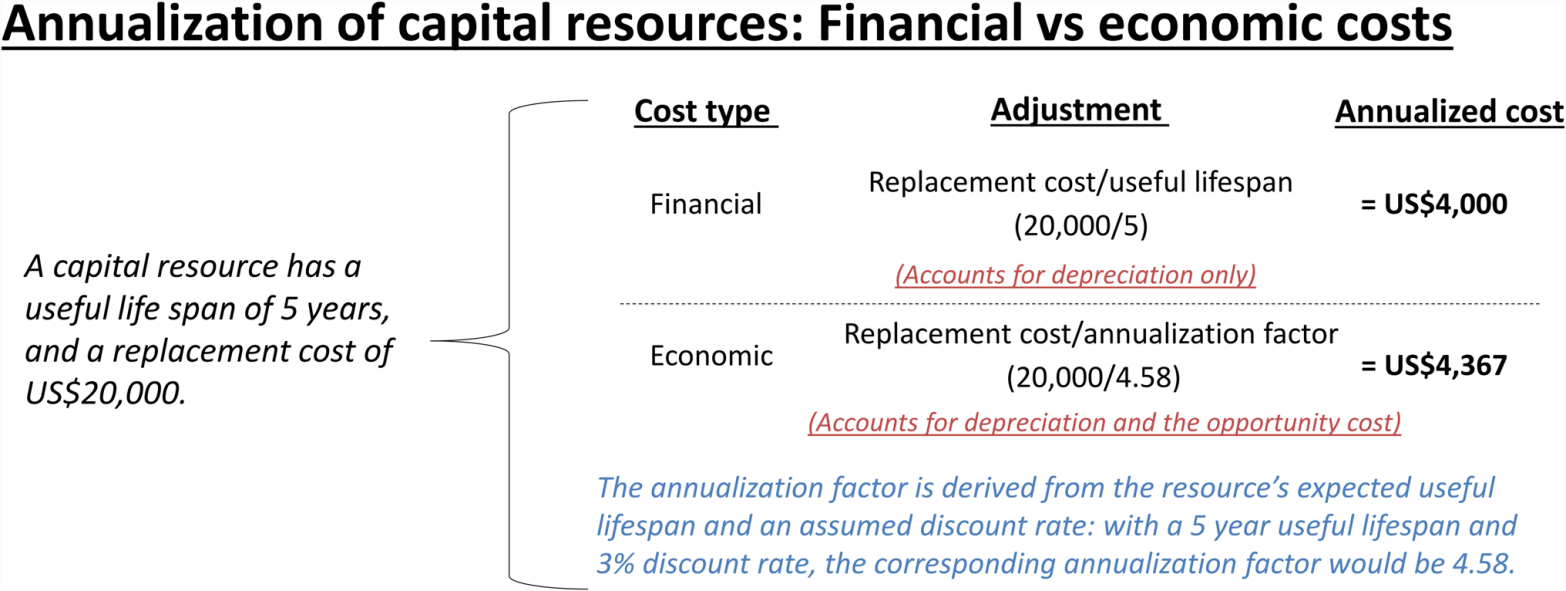
The difference between the annualized financial and economic costs of a capital resource*.* As capital resources (such as vehicles) are bought in 1 year but used over several years, their cost needs to be spread over their useful life. This adjustment is known as annualization, and it has two potential components: depreciation (the reduction in the value of the asset over time due to wear and tear) and the opportunity cost associated with tying up the funds in purchasing the capital item (as there is a lost opportunity to generate gains from investing that capital). When calculating financial costs, the annualization calculation only captures depreciation, by dividing its replacement cost by its useful lifespan). In contrast, when calculating economic costs, the annualization calculation also aims to capture the opportunity cost. This is done by dividing the replacement cost by an annualization factor, which is based on the resource’s expected lifespan and an assumed discount rate. Because the annualization factor is a smaller number than the corresponding resource’s expected lifespan (here 4.58 versus 5), the annualized economic cost will be higher than the annualized financial cost. For further details, see Walker et al. [67]. Figure adapted from ref. [14]

Financial costs represent the actual financial outlays for the goods, resources, and services that are purchased [11, 14]. In this context, the financial cost of an intervention represents the amount of money that was paid for the resources being used, and they are typically based on or similar to expenditure data [11, 14]. However, a difference between financial costs and expenditure data is that financial costs also capture the depreciation in value of capital resources (inputs that can be used for more than 1 year, such as equipment and vehicles) over time through annualization (Fig. 2) [11, 14]. The terms fiscal cost/undepreciated financial cost/expenditures are used to represent financial costs without accounting for the depreciation of capital costs [32, 66]. These expenditures reflect the financial outlay that an agent (e.g., government, donor, or individual) spends during a period of time for goods and services, with all capital goods expenditures being recorded in full as they are incurred and not annualized [11].

Economic costs represent the full value of the resources utilized in providing an intervention [11, 14]. Crucially, they are intended to capture the resources’ opportunity cost, and they are based on the value of the resources’ next-best alternative use that has been forgone owing to them being utilized and not simply the monetary amount paid for them. Consequently, with economic costs, all relevant resources consumed by an intervention should be valued, not just those constituting a budgetary line or expenditure. This is a broader conceptualization of a resource’s value than its financial cost, and this framework recognizes both that resources can have a value that may not be fully captured by the price that has been paid for it and that by using a resource it makes it unavailable for productive use elsewhere [14]. Further details on the differences between financial and economic cost calculations are outlined by Turner et al. [14].

### Fixed versus Variable Costs

Fixed costs are those costs that do not vary with scale (i.e., they do not change with the level of output). For example, these costs would not change depending on the number of patients that are reached by an intervention and would be incurred even if the output were zero. Common examples of fixed costs include buildings and equipment. In practice, these will often be used for multiple interventions (see “shared costs”)—therefore, only a proportion of the asset that is being used is allocated to the intervention rather than the entire cost of the asset.

Variable costs are those that vary with scale (i.e., they change with the level of output of an intervention). For example, these costs would change depending on the number of patients that are reached by an intervention (such as costs related to consumables or drugs).

Which costs are fixed (or even the level at which they are fixed) depends on context. Note that it is possible to have stepped-fixed costs that are fixed for a particular level of activity/production but increase incrementally once a threshold is crossed. For example, the number of staff that would be required to screen for a particular disease at a school would be influenced by the number of children that are being sampled at each school and would increase incrementally depending on the number each staff member could screen [68].

### Capital versus Recurrent Cost

Capital (or one-off) costs are one-time costs for resources (or activities) that have a useful life of more than 1 year—such as buildings, vehicles, or medical equipment [11]. As these resources are bought in 1 year but used over several years, their cost needs to be spread over their useful life. This adjustment is known as annualization (also known as amortization), and the calculation is different for financial versus economic costs (Fig. 2). Note that start-up costs (cost of initial one-time programmatic activities—outlined further in Sect. 4) or even trainings can also be treated as capital costs.

Recurrent costs are the costs for resources/inputs with useful lives of less than 1 year, such as costs related to supplies and personnel [32].

### Charge, Reimbursement Rates and Cost-to-Charge Ratios

When using data related to hospitals or clinics, it is important to consider the difference between charges, reimbursement rates, and true costs in an economic sense.

Charges/fees represent the amount of money a service provider (such as a hospital or clinic) “bill” for their services to a patient—i.e., what is on the patient’s bill from a hospital [12]. Crucially, charges do not necessarily reflect the true value or the actual cost of the resources used.

In contrast, a reimbursement rate or tariff is a set monetary amount that healthcare systems pay a health service provider for particular categories of activity/tasks (such as the amount of money a hospital would receive from a governmental social health insurance program for performing a particular diagnostic test). These reimbursement rates or tariffs will not necessarily be the costs actually incurred by the healthcare sector/provider or even the same as what the service provider charges the patients in fees. Note that the term reimbursement rate can also be used to describe the amount an insured person is reimbursed by their insurance company after having incurred a healthcare-related expense.

It should be noted that healthcare providers’ revenue can be generated from several different sources (potentially with capital costs being financed separately) [12]. Moreover, hospitals and large outpatient centers are complex, multiproduct/multidepartment organizations, where cross-subsidization can be notable [12]. Therefore, charges/tariffs/fees should be used cautiously as proxies of provider costs, especially if there is an indication that they may not be reasonable estimates of actual provider cost [12].

To more accurately capture economic costs within this context, a cost-to-charge ratio is commonly applied to charge or reimbursement rate data. This can be based on the ratio of the hospital’s (or a specific department’s) total expenses versus the money brought in via charges [69, 70]. This ratio is usually determined by special costing studies [12]. It should be noted that such ratios will be an approximation, since there will rarely be enough detail to determine different ratios for different procedures/types of patients.

What type of data is appropriate to use for these types of cost items will depend on the perspective of the analysis; with charge-based data more appropriate under the patient perspective, reimbursement rate-based data under a specific payer perspective, and the approximated true cost under the health system/sector/provider and societal perspectives [71]. If charge-based data or reimbursement rates/tariffs are used to represent the true costs under the health system/sector and societal perspectives without adjusting with a cost-to-charge ratio, it should be stated clearly as a limitation.

## Other Cost Terms

### Out-of-Pocket Payments

Out-of-pocket payments refer to spending directly borne by an individual/household for health services that are not reimbursed by any third party (such as a health insurance program) [18]. The level of out-of-pocket payments that patients face can vary significantly depending on the type of healthcare system, the specific health insurance plan, and the individual’s health status.

Out-of-pocket payments are a key component of catastrophic health expenditure calculations and are relevant to discussions around financial risk protection [18]. These can be inconsistently defined within the literature, with some studies focusing only on direct medical costs and others also including direct nonmedical or even productivity costs within this category. We would recommend being explicit regarding what types of costs are included.

### Transfer Costs

Transfer costs or payments are financial flows from one part of the healthcare system or society (depending on the perspective) to another that do not consume resources but simply transfer the power to use resources from one person or sector to another (such as import tariffs as well as unemployment or sickness benefits) [22]. Transfer payments can be a cost to the paying government or control program, but a financial gain to another sector or a patient (such as import tariffs or sickness benefits [20]). The inclusion or exclusion of transfer costs will depend on the perspective of the study [24]; as transfer costs do not use or create resources, they are not typically considered when estimating economic costs when using a societal perspective [35, 36].

### Intangible Costs

Intangible costs describe costs that cannot be directly quantified in monetary terms (such as those related to pain or depression as a result of illness or treatment) [16, 17]. These have to be valued monetarily indirectly, such as based on willingness to pay approaches (i.e., estimating how much the patient would pay not to have their pain). These are rarely included in costing studies [72]. It should be noted that this term is used inconsistently and is also sometimes used as an umbrella term describing costs that are difficult to measure [12].

### Shared Costs

Shared (joint) costs are related to resources that are jointly used between different interventions [20]. These shared costs are allocated according to the proportion of their use between the different interventions. For example, if a vehicle was being used by multiple control programs, its costs would be allocated on the basis of its relative use. The methods used to allocate shared resources should reflect usage of each input and should be explicit [11].

In particular, careful attention should be paid to methods used to allocate human personnel-related costs [11]. Importantly, there is no “gold standard” approach, and the different methods (group discussions, interviews, examining patient records, time sheets, etc.) each have their own biases/limitations (further discussed in ref. [11]).

More specific methodological guidance for allocated shared costs (focusing on vaccination programs) is outlined by Brenzel [28].

### Start-Up Costs

Start-up costs relate to initial one-time programmatic activities, such as initial training activities, and initial sensitization/social mobilization [21]. Any routine or repeated programmatic activities would not fall under this category. Start-up activities are defined by the nonrepeating nature of the activity, not the type of input [21]. Note that start-up costs can be treated as capital items and annualized.

### Overhead Costs

Overhead costs refer to costs that cannot be directly traced to the provision of a service (such as administration, security personnel, buildings, etc.) [11]. In some texts, these costs are referred to as indirect costs. To avoid confusion with the terminology, the Global Health Cost Consortium Reference Case recommended referring to these as “operational” activity costs [11].

### Sunk Cost

Sunk costs are costs that have already been incurred and that cannot be retrieved [11]. These should generally be ignored when considering economic costs because they will remain the same regardless of the outcome of a decision, and what resources have been used in the past is not a determinant of the optimum decision of how to allocate resources moving forward [34]. However, it is important to note that there can be ongoing opportunity costs related to previously purchased resources (such as the use of building space or vehicles) if they could be used for other services within the timeframe of the study [14].

### Tradable versus Nontradable Resources

Tradable resources are those that can be sold in a different country from that in which they are produced (such as laboratory equipment and drugs) [73]. Nontradable resources are those that cannot be exported or imported, i.e., they cannot be traded on the international market and are only be bought/consumed in the country in which they are produced (in general, local personnel, utilities, facilities/land, and domestic transport are treated as nontraded goods) [73].

### Future Costs—Related and Unrelated

Future related medical costs are medical costs occurring in the future that are directly related to the disease under investigation. For example, an intervention to treat heart disease will extend a patient’s life, but there will be future costs associated with routine visits to a cardiologist, for example [15]. This can also include related medical costs that are averted in the future (for example, successful treatment of hepatitis C can avert the need for a liver transplant in the future if the disease is left to progress). Averted future medical costs are particularly relevant for preventive interventions.

Future unrelated medical costs are medical costs that are unrelated to the disease being investigated. For example, an intervention may extend a patient’s life, but they may then subsequently incur other medical costs associated with unrelated conditions (e.g., 3 years after a successful cancer treatment the patient develops diabetes) [15]. Future costs that are not related to medical resources can include the monetary value of the productivity gains associated with an intervention [15]. For example, if a patient is treated and survives a heart attack, they are then able to return to work.

There has been considerable debate regarding the types of future costs that should be included within health economic studies [15, 74, 75]. This is particularly relevant regarding the inclusion of future unrelated medical costs [15], which depend on unrelated events occurring in the future that are affected by decisions not yet made and are therefore difficult to predict/include. This debate and variation should be considered when interpreting different studies.

## Cost Measures

### Average (Unit) Cost, Marginal Costs, and Incremental Costs

The average (unit) cost measure describes the total cost per unit of output of an intervention. These “unit” costs can be measured across a whole program or for a specific site/area [11]. Average (unit) costs can include all the costs involved in producing a service compared with doing nothing.

In contrast, the marginal cost measure describes the cost of producing one or more unit(s) of a service/output of an intervention (such as the cost of treating an additional person). This captures how additional costs change as service/output levels increase one unit at a time [11]*.* One key difference between the quantification of average (unit) costs and marginal costs is that fixed costs are not considered within marginal cost calculations.

The incremental cost term can describe the difference in cost between two or more mutually exclusive interventions or strategies (such as treatment with different drugs) or compare the cost associated with a change of scale or approach to an intervention (such as providing the intervention at a higher coverage or the cost of adding a new service to an existing program) [11, 21]. It should be noted that the terms marginal and incremental costs are sometimes used interchangeably, as they both can refer to a change in the scale of an activity [11]. The difference between them is that incremental costs represent the cost difference between two alternative strategies irrespective of the change in the volume of service output, whereas marginal costs represent the additional costs of producing an additional unit of output.

Which cost measure should be applied in cost estimation depends on the specific research question. For further guidance, look at the Global Health Cost Consortium Reference Case for estimating the costs of global health services and interventions [11]. Incremental costs have an important role within economic evaluations.

## Cost Adjustments

### Inflation Adjustments

It is often necessary to use cost data that have been collected at different time points [73]. However, owing to inflation, the purchasing power of a currency often decreases over time; it often costs progressively more to provide the same quantity of goods/services. Owing to inflation, costs from different years are not directly comparable with each other. Within health economic studies it is often necessary to adjust costs obtained from different time periods to express them in a single base or reference year. Without making such an adjustment, it can be difficult to tell whether a change in the cost of an intervention over time is due to a change in the real value of the resources being used or to a change in the value of the currency used to purchase them. For example, if we quantified the costs of an intervention from 2010 to 2015, we would adjust the costs incurred between 2010 and 2014 to 2015 prices (i.e., 2015 would be the base year of the analysis), making the costs comparable. It is important that the currency year and exchange/conversion rate are clearly reported.

Inflation adjustments can be performed with a relatively straightforward calculation using GDP deflators or the consumer price index (CPI) [11, 73]. That said, the different approaches that can be used can have a notable difference on the outcome (such as the use of inflation rates relating to US dollars or the local currency). A key factor that influences the most appropriate approach relates to whether the resource/good in question is tradable or nontradable [11, 73]. For nontradable local goods, it is preferable to inflate in the local currency and then adjust to US dollar using the corresponding exchange rate for the base year [11, 73]. However, for tradable inputs (which will include many global health commodities e.g., testing machines and antiviral drugs), GDP deflators or the CPI do not necessarily capture price changes well; many global health commodities will actually have decreasing prices over time [11]. For these tradable goods, where feasible, it is preferable to update the cost using the commodity-specific price change if the current prices are available [11]. If this is not feasible, the cost of the goods can be inflated using the US dollar GDP deflator*.* More information on the methods for adjusting for inflation are outlined within Turner et al. [73].

### Discounting

Discounting is the process used to adjust the costs (or benefits) occurring in the future to their present value, i.e., into the equivalent value that those costs or benefits would have now [76–78]. It aims to reflect the view that people generally prefer to receive benefits or goods now but pay for them later (i.e., a “time preference”). Discounting also reflects the opportunity cost associated with spending money now when instead it could alternatively be invested to generate a return in the future. Consequently, costs incurred in the present are of greater importance than costs incurred in the future. Discounting allows for the comparison of the costs/outcomes occurring over different time periods.

It is important to clarify that although discounting costs and adjusting for inflation are mathematically similar (and sometimes confused), they are different processes [73]. Adjusting for inflation is about accounting for how the purchasing power of a currency has changed over time, whereas discounting future costs is about accounting for the opportunity cost associated with spending money now rather than in the future and the societal time preference that results in costs incurred in the present being of greater importance and value than costs incurred in the future [73, 76–78].

**Fig. 3:**
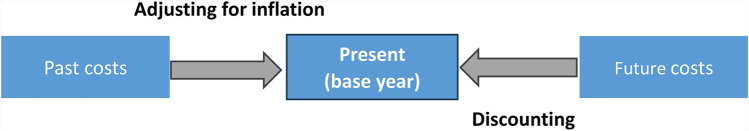
Discounting versus inflation in the context of an economic evaluation

In the context of performing an economic evaluation, any cost inputs from the past are typically adjusted for inflation so that they are expressed in a single base year. In contrast, the costs projected to occur in the future would be discounted from the base year onward (Fig. 3).

### Currency Changes

It can be necessary to adjust to different currencies within health economic analysis [73]. Within a global health context, it is common to report costs in US dollars (although it can be useful to report the costs in the local currency as well). This conversation is typically done using a market exchange rate—which determines a currency’s value in relation to other currencies. It can be important to consider currency changes when adjusting for inflation [73].

### Purchasing Power Parity and International Dollars

Different currencies have different purchasing powers. To account for this, some studies use international dollars (I$)—a hypothetical currency unit that is designed to capture the differences in relative prices across different settings [20, 79, 80]: I$1 would buy in the country of interest a comparable amount of goods and services as US$1 in the USA. International dollars can be used to compare the cost of goods and services in different countries on a like-to-like basis [20]. Costs in the local country currency units are converted to international dollars using purchasing power parity (PPP) exchange rates (which are available from the World Bank’s World Development Indicators Databank [81]). The choice of whether to present costs in US dollars or international dollars will depend on the aim of the study and its setting(s) [73]. These options are not mutually exclusive, and the same study can report the results with different currencies. Studies adjusting using international dollars as their main output should clearly indicate this and differentiate it from US dollar output (i.e., indicating values adjusted to international dollars (such as I$) and not simply $, US$, USD, etc.).

## Costs are Not Constant: the Importance of Economies of Scale and Scope

The total cost of an intervention can consist of both fixed and variable costs, and therefore the average (unit) cost will not necessarily be constant at different levels of output. This is because the fixed costs do not change at different levels of output, which therefore changes the unit cost at different levels of output [82–84]. The degree of this change depends on the proportion of fixed versus variable costs.

**Fig. 4:**
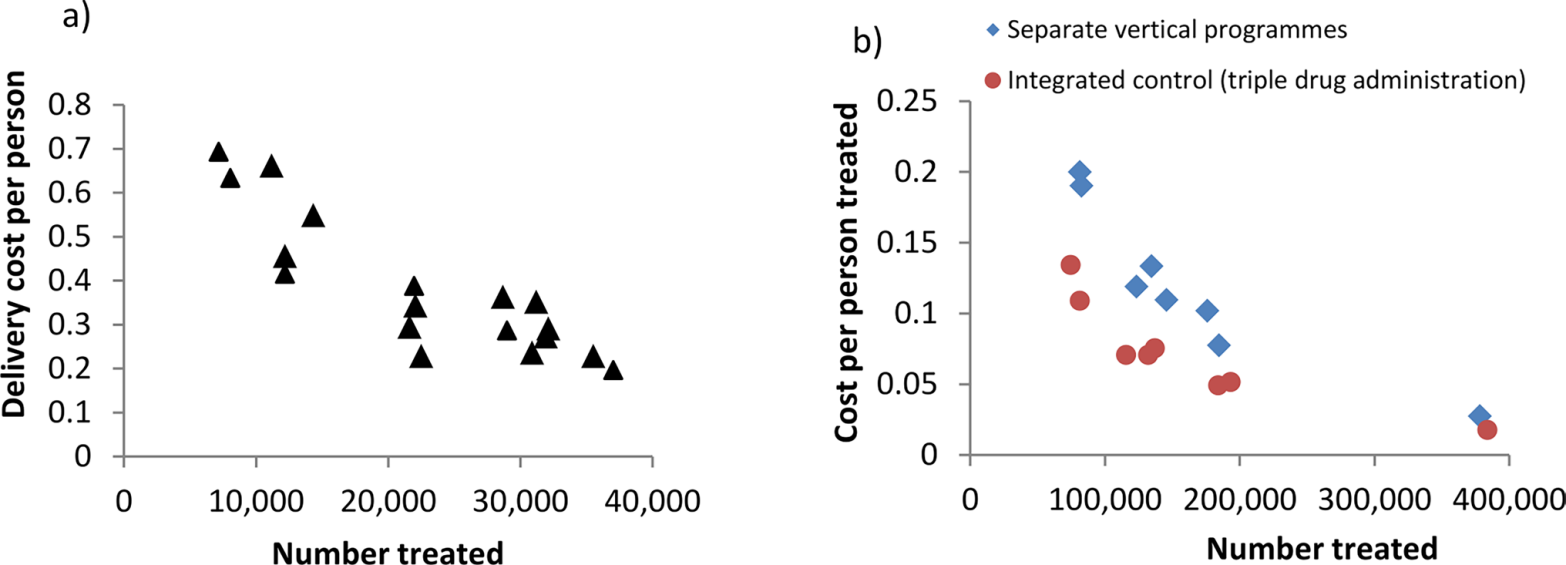
Economies of scale and scope case study related to mass drug administration. Data from **a** Brooker et al. [88] and **b** Evans et al. [89]. Figure adapted from ref. [90]

This can result in “economies of scale,” where there is a reduction in the average cost per unit of an intervention resulting from increased production/output [11]. An example of this is shown in Fig. 4, where there is a reduction in the unit cost per treatment as a result of increasing the number of people treated. This occurs because, at low levels of production, fixed costs are spread across a low number of outputs, and so the unit cost is relatively high. As the level of output increases, the fixed costs are spread across more outputs and unit cost decreases. An average cost function can be used to describe how unit costs vary as the level of intervention/service increases [11]. Average cost functions exhibit different shapes. In some circumstances, unit costs remain constant for all levels of service provision. However, unit costs often vary (even nonlinearly) as the level of the output of an intervention/service changes [11]. At a certain level, unit costs may also begin to increase or exhibit “diseconomies of scale”—where the unit costs start to increase as the output increases. This could be because the service is becoming more complex/harder to run and/or owing to expanding into harder-to-reach areas. Theoretically, this can result in a U-shaped cost curve—where initially there are “economies of scale” followed by “diseconomies of scale.” However, although this can occur [6, 85], there is a scarcity of literature supporting this quantitatively [86, 87], and it will depend on the context, i.e., the type of intervention, targeted population, and the setting. In practice, the patterns of how costs change with scale can be complex, and it is important to consider scale-dependencies when generalizing cost data.

A further issue to consider is that the unit cost of an intervention or service can vary depending on the other services it is delivered with. “Economies of scope” can exist where the average (unit) cost is reduced when multiple services are delivered jointly (such as when integrating the treatment of diseases rather than using separate vertical programs) (Fig. 4).

In summary, an intervention’s unit costs can be influenced by both the level of output and the scope of service (Fig. 4), both of which have the potential to change over time. It is therefore important that when using “unit costs”, it is understood that that cost may only be a good estimate in its corresponding context/scale [11]. While in some cases economies of scale and scope may not have a significant role, and the unit cost for a service/intervention can be characterized by a single unit cost value, this will not always be the case, particularly for large-scale preventive interventions [11].

In practice, some sort of empirical validation/fitting to data would typically be required before choosing a specific form of cost function [11]. Where this is not possible, the use of a single unit cost is generally accepted for most costing purposes [11]. d’Elbée et al. [91] conducted a review of this area and developed new mathematical notations and cost function frameworks for the analysis of healthcare costs at scale in LMIC settings.

## What to do in Practice?

### Determining the Need for a New Costing Study

Before embarking on a new costing study, researchers should first assess whether it is truly necessary. The first step is to review available evidence and databases to see if relevant cost data already exist. In many cases, previous studies or government reports may have already estimated similar costs, making a new study redundant. However, having cost data available is not enough—researchers must also assess its relevance. This means considering whether the data applies to the specific research question, population, and healthcare setting. Differences in healthcare infrastructure, service delivery models, or economic conditions across regions may limit the applicability of existing estimates.

Beyond relevance, the quality and credibility of the data must also be carefully examined. Cost estimates should be derived from methodologically sound studies with transparent reporting. Existing data sources may not be suitable for use if they have significant biases, missing information, or outdated cost structures. Researchers should also ensure that the relevant cost components are included that align with the study’s objectives.

If existing cost data are of acceptable quality, and fit for purpose, conducting a new costing study may not be necessary. That said, there are situations in which a new study will add value/be needed. Ultimately, the decision to conduct a new costing study should be guided by whether it adds meaningful value to the available evidence. Note that this will often be more of a trade-off rather than a black-and-white decision, and the extra cost and time of a new costing study need to be justifiable on the basis of the improvement that the study will bring to cost estimates. By carefully considering relevance, quality, and fit-for-purpose, researchers can ensure that resources are used efficiently and that cost estimates truly support informed decision-making. If gaps in cost data exist, alternative approaches—such as expert consultations, data extrapolation, or sensitivity analyses—can sometimes provide sufficient estimates without the need for a full-scale costing study. The Guide to Economic Analysis and Research (GEAR) online resource also provides guidance to researchers on identifying relevant cost data when there is limited information, particularly from LMICs [92].

### Type of Costing Approach and Source of Cost Data

The methodological approach taken to a costing study will depend on the following three factors: (1) The purpose/objective/intended policy use of the cost data which is being collected, (2) availability of the data and its sources in the country context, and (3) level of resources/the feasibility.

In practice, a combination of approaches may be needed (for example a microcosting study may use both bottom-up and top-down methods [42]). The framework presented by Hendriks et al. [93] also highlighted that the use of mixed methodology can be used to account for limited data availability and/or limited relevant expertise/capacity—which can be particularly relevant to LMICs.

**Fig. 5:**
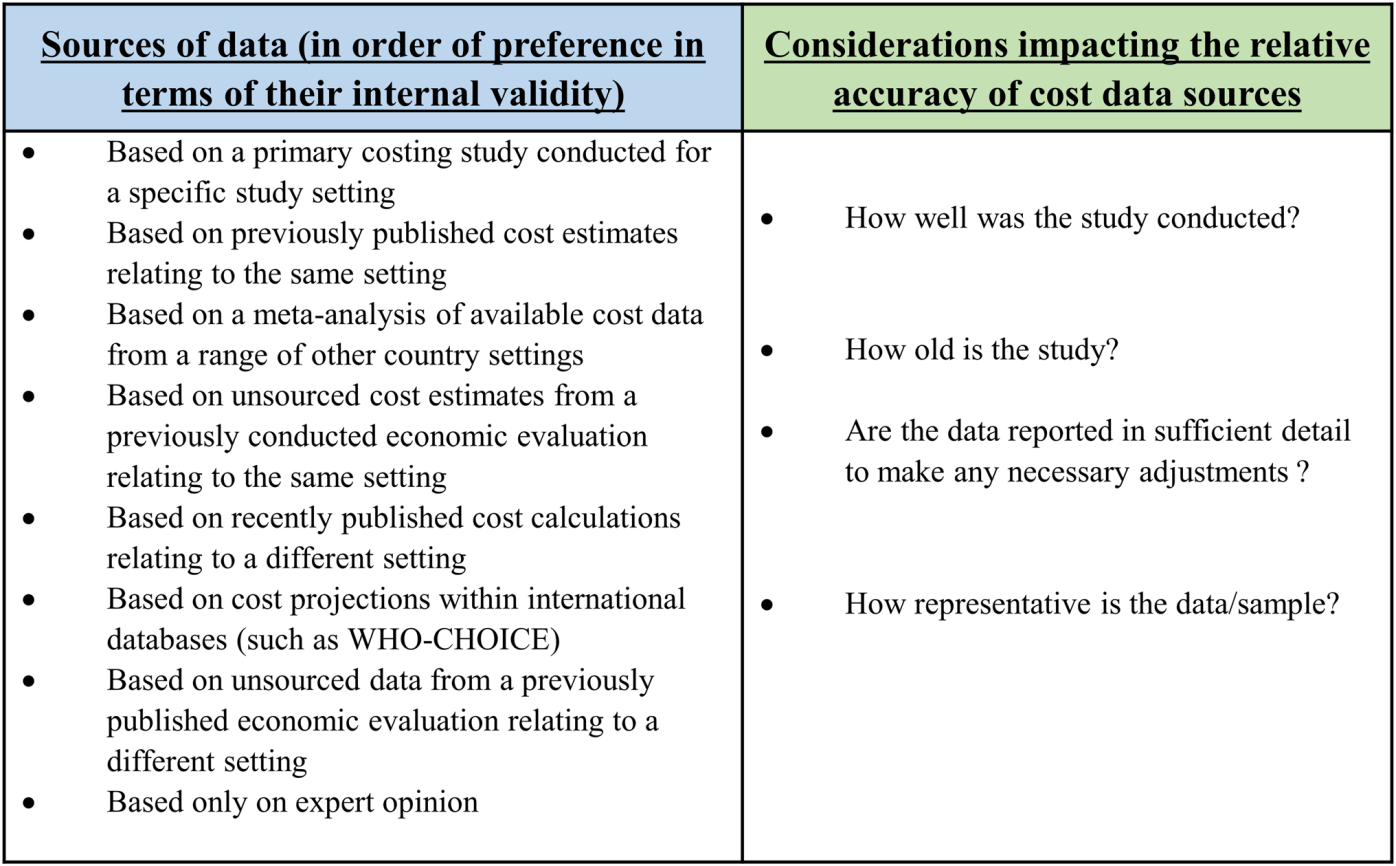
Potential sources of cost data and considerations impacting their relative accuracy. Ultimately, the source that is the most accurate will depend on the context of the study, and any hierarchy of cost data sources should be applied flexibly

The source of cost data will be influenced by the local context and data availability. Note that all sources of cost data are not equal. Figure 5 shows the different potential sources of cost data and considerations regarding their relative accuracy. Ultimately, the source that is the most accurate will depend on the context of the study, and any hierarchy of cost data sources should be applied flexibly.

In some countries (such as the Netherlands, Thailand, and India), a potential source of cost data is their standardized cost menus, which represent the standard costs for medical services in a particular country [94–96]. International databases also exist that are specific to certain interventions (such as the Immunization Delivery Cost Catalogue [97]).

### Type of Costs to Use/Collect

The type of cost that should be used will depend on the purpose of the study. For example, financial costs are needed for the purposes of budgeting and planning of health services, as well as understanding the affordability of a new intervention (such as within a budget impact analysis [98]). In contrast, economic costs are needed when assessing the value for money of alternative policy options for informing policy decisions, i.e., making choices regarding the allocation of scarce healthcare resources [14]. The Global Health Cost Consortium Reference Case [11] outlines in more detail recommendations regarding what cost measure should be used and when.

What cost inputs need to be collected will be linked to the perspective to be used for the study, such as societal, healthcare provider, etc. [24]. Generally, in terms of the cost of the intervention, the broader the perspective, the more costs would be included and the higher the potential cost of the intervention—although the impact of broadening the perspective will depend on the context and the cost will not necessarily increase [24]. For example, when looking at the costs of providing a vaccine at a health clinic, the healthcare provider(s) perspective would only include the costs that are incurred by the government’s health service (such as those associated with the staff’s time, the purchase of the vaccine, etc.). However, under the societal perspective, the direct nonmedical costs that are incurred by the patients to travel to the clinic and get the vaccine would also be included and potentially their productivity costs associated with lost paid or unpaid work, increasing the overall cost of the intervention.

### Reporting and Critical Appraisal

In terms of reporting the results of costing studies, we recommend following the principles and methods reporting checklist developed within the Global Health Cost Consortium reference case—which is comprehensive and relates to 17 key areas across four groups; namely, study design and scope, service and resource-use measurement, valuation and pricing, and analyzing and presenting results [11]. The Consolidated Health Economic Evaluation Reporting Standards (CHEERS) checklist [99] also provides comprehensive guidance on reporting economic evaluations, including categories for cost measurement and valuation. In addition, quality assessment tools and frameworks—such as the Consensus on Health Economic Criteria (CHEC) checklist by Evers et al. [100]—provide a structured approach for evaluating the methodological quality of economic evaluations and include key categories related to costing. Furthermore, Schnitzler et al., [101] have developed a consensus-based checklist specifically designed for the critical appraisal of cost-of-illness studies.

Ensuring high-quality reporting in this area not only enhances the ability of policymakers to interpret and utilize cost data for decision-making but also provides significant value to future research. Transparent and well-documented cost data allow other researchers to build upon previous studies, facilitating comparative analyses, meta-analyses, and methodological advancements in economic evaluation. By ensuring consistency and rigor in cost reporting, researchers contribute to a more robust evidence base that can improve the generalizability and applicability of cost findings across different settings.

Verifying and validating cost estimation in health economic evaluations is crucial to ensuring accuracy, credibility, and policy relevance. Similar to model validation, cost validation should be conducted at both the disaggregated and aggregated levels. At the disaggregated level, individual cost components should be examined for consistency, plausibility, and appropriateness of data sources. At the aggregated level, total cost estimates should be cross-checked with other sources to assess their validity. Validation can be performed internally through face validation, where stakeholders—including policymakers, healthcare providers, and economists—review and verify cost estimations.

## Declarations

### Availability of Data and Materials

The authors confirm that the data supporting the findings of this study are available within the article.
